# Targeting mutant estrogen receptors

**DOI:** 10.7554/eLife.44181

**Published:** 2019-01-16

**Authors:** Suzanne E Wardell, John D Norris, Donald P McDonnell

**Affiliations:** Department of Pharmacology and Cancer BiologyDuke University School of MedicineDurhamUnited States

**Keywords:** breast cancer, acquired drug resistance, hormone therapy, bazedoxifene, estrogen receptor, SERM/SERD, Human

## Abstract

A drug used in hormone replacement therapy can target estrogen receptors that have become resistant to breast cancer treatments.

**Related research article** Fanning SW, Jeselsohn R, Dharmarajan V, Mayne CG, Karimi M, Buchwalter G, Houtman R, Toy W, Fowler CE, Han R, Lainé M, Carlson KE, Martin TA, Nowak J, Nwachukwu JC, Hosfield DJ, Chandarlapaty S, Tajkhorshid E, Nettles KW, Griffin PR, Shen Y, Katzenellenbogen JA, Brown M, Greene GL. 2018. The SERM/SERD bazedoxifene disrupts ESR1 helix 12 to overcome acquired hormone resistance in breast cancer cells. *eLife* **7**:e37161. doi: 10.7554/eLife.37161

In three quarters of human breast cancers, the tumor cells have estrogen receptors and grow when exposed to this hormone. Most of these cancers will respond to drugs that target the activity of the receptor. For example, selective estrogen receptor modulators (SERMs) work by competing with estrogen and taking the hormone’s place on the receptor. Selective estrogen receptor downregulators (SERDs) behave in a similar way, but they can also trigger the degradation of the receptors.

The first breast cancer therapy targeting estrogen receptors to be approved in the United States was a SERM called tamoxifen, yet drugs that inhibit the production of estrogen have fewer side effects and are usually the first choice when treating patients with post-menopausal breast cancer. However, recent data suggest that mutations in estrogen receptors can reduce the effectiveness of these drugs. Such mutations are rare before cancer treatment, but they are found at a much higher rate in patients in which the disease has progressed (Toy et al., 2013).

The mutations most commonly associated with cancer spreading, Y537S and D538G, occur within the helix 12 subdomain, a region of the receptor that is exceptionally important for ligand pharmacology. The resistance caused by these mutations limits the therapeutic lifespan of cancer treatments, and highlights the importance of developing new SERMs and SERDs which can be used against tumors that stopped responding to drugs.

When estrogen binds to a wild-type receptor, the helix 12 subdomain folds and clamps the hormone in the ligand-binding pocket of the receptor (Figure 1A). At the same time, this change creates an interacting surface for coactivators, the proteins that cooperate with the receptor to activate gene transcription (Celik et al., 2007). However, the binding of a SERM changes the shape of the helix 12 subdomain in a way that inactivates the receptor by preventing its interaction with a coactivator. When a SERD occupies the receptor, the resulting modification of the helix 12 subdomain leads to the degradation of the receptor.

**Figure 1.**
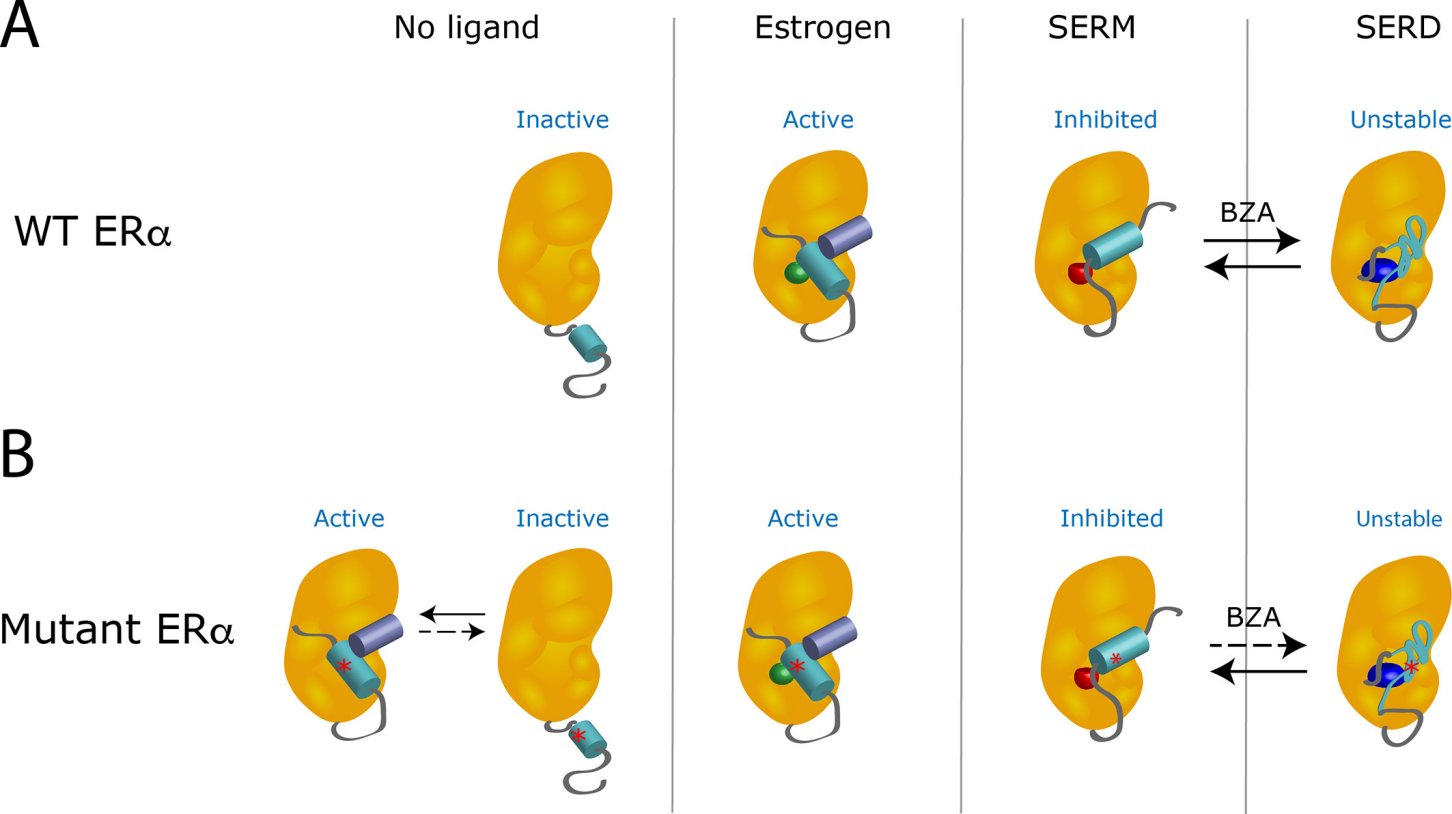
The Y537S mutation stabilizes the estrogen receptor and interferes with the action of drugs. (**A**) In the absence of a ligand, the helix 12 subdomain (blue-green cylinder) of the wild-type estrogen receptor (WT ERα, orange structure) is flexible. As the receptor binds estrogen (green sphere), the helix 12 subdomain folds in a way that allows coactivator proteins (purple blue cylinder) to attach to the receptor. When the ligand-binding domain is occupied by a SERM (red sphere), the helix 12 subdomain is repositioned and occludes the activation surface, which inhibits the receptor. Treatment with a SERD (dark blue ovoid) destabilizes the helix 12 subdomain, leading to the degradation of the receptor. When the receptor is exposed to hybrid SERM/SERD molecules, such as bazedoxifene (BZA), the helix 12 subdomain can adopt either SERM or SERD conformations (black arrows): the receptor is continuously inhibited, even though it is inefficiently degraded. (**B**) The mutation Y537S (asterisk) stabilizes the helix 12 subdomain in the active conformation, which activates the receptor even in the absence of estrogen (left). When treated with a SERM or SERD, the mutant receptor can adopt conformations similar to the ones observed when the wild-type receptor is bound to these drugs. However, the mutation lowers the affinity of the mutant receptor for SERMs and SERDs; even after treatment with these drugs, unliganded receptors that are active can be present. The SERM/SERD hybrid drug bazedoxifene has a high enough affinity that it can bind to and fully inhibit the mutant receptor, making it adopt the SERM-associated conformation. However, it does not efficiently cause the mutant receptor to adopt the SERD conformation (dotted arrow); this can therefore result in the mutant receptor being inhibited, but not degraded. Image credit: Suzanne E Wardell (CC BY 4.0).

Experiments also showed that Y537S and D538G mutations stabilize the unliganded receptor, and allow the helix 12 subdomain to adopt an active conformation: the mutant receptors are activated even in the absence of estrogen (Fanning et al., 2016; Figure 1B). These mutations also reduce the affinity of the receptors for their hormone and for most SERMs and SERDs. Therefore, even in the presence of the SERMs or SERDs currently used in breast cancer treatment, the receptor favors the active conformation. Now, in eLife, Geoffrey Greene of the University of Chicago and colleagues in the US and the Netherlands – including Sean Fanning as first author – report how a hybrid SERM/SERD drug can overcome the resistance conferred by mutant receptors (Fanning et al., 2018).

Hybrid SERM/SERD molecules, such as the drug bazedoxifene, display exceptional inhibitory activities in tumor models, but they degrade estrogen receptors very inefficiently. Fanning et al. address this long-standing paradox, and demonstrate that bazedoxifene allows the helix 12 subdomain to align in two distinct orientations: one replicates the inactive conformation observed when the receptor binds to a classical SERM, and the other mimics the unstable structure found when a classical SERD occupies the receptor. However, estrogen receptors with the Y537S and D538G mutations are more stable: when they are bound to bazedoxifene, they primarily adopt the SERM-associated conformation, rather than the one observed with SERDs. Although the mutant receptor is then not degraded, it remains efficiently inhibited in this inactive conformation.

Bazedoxifene is a well-characterized modulator of estrogen receptors, and it is currently used in hormone replacement therapy and to treat post-menopausal osteoporosis (Kawate and Takayanagi, 2011; Komm et al., 2014). While recently developed SERDs have struggled with clinical setbacks due to intolerability, this drug has been thoroughly tested in patients and is both safe and tolerable. Intense efforts are underway to identify new drugs that target estrogen receptors, and some interesting new molecules have already emerged, but they are not likely to be approved for clinical use for several years. Prior studies have shown that bazedoxifene can reduce estrogen receptors within tumors, albeit with less efficiency than other benchmark SERD drugs. Bazedoxifene can also inhibit the growth of tumors derived from patients that carry mutations in their estrogen receptors (Wardell et al., 2013; Wardell et al., 2015). These data raise the possibility that the compound might be of some clinical utility in breast cancer treatment.

The work by Fanning et al. describes the mechanisms by which bazedoxifene inhibits estrogen receptors, and offers guidance for the development of new treatments for breast cancer. Although the drug is likely to help control cancers that have progressed during treatment, independent of mutant status, its utility may be limited by patent life; there clearly remains an unmet medical need for new molecules that can effectively inhibit and degrade clinically relevant mutations of the receptors.
